# Neuropsychological Symptom Identification and Classification in the Hospitalized COVID-19 Patients During the First Wave of the Pandemic in a Front-Line Spanish Tertiary Hospital

**DOI:** 10.3389/fpsyt.2022.838239

**Published:** 2022-03-02

**Authors:** Juan D. Molina, Irene Rodrigo Holgado, Alba Juanes González, Carolina Elisa Combarro Ripoll, David Lora Pablos, Gabriel Rubio, Jordi Alonso, Francisco P. J. Rivas-Clemente

**Affiliations:** ^1^Centro de Investigación Biomédica en Red (CIBER) Salud Mental, Madrid, Spain; ^2^Villaverde Mental Health Center, Clinical Management Area of Psychiatry and Mental Health, Psychiatric Service, Hospital Universitario 12 de Octubre, Madrid, Spain; ^3^Research Institute Hospital 12 de Octubre (i + 12), Madrid, Spain; ^4^Faculty of Health Sciences, Universidad Francisco de Vitoria, Madrid, Spain; ^5^Clinic Psychologist, COVID-19 Assistance Project, Psychotherapy Unit, 12 de Octubre University Hospital, Madrid, Spain; ^6^Psychiatrist, COVID-19 Assistance Project, 12 de Octubre University Hospital, Madrid, Spain; ^7^CIBER de Epidemiología y Salud Pública, Madrid, Spain; ^8^Faculty of Statistics, Complutense University of Madrid, Madrid, Spain; ^9^Department of Psychiatry, Faculty of Medicine, Complutense University of Madrid, Madrid, Spain; ^10^Addictive Disorders Network, Redes Temáticas de Investigación Cooperativa (RETICS) (Thematic Networks of Cooperative Research in Health), Carlos III Health Institute, Ministerio de Ciencia e Innovación (MICINN) and Federación Española de Enfermedades Raras (FEDER), Madrid, Spain; ^11^Institut Municipal D'Investigacio Medica (IMIM)-Hospital del Mar Medical Research Institute, Pompeu Fabra University, Barcelona, Spain; ^12^Centro de Investigación Biomédica en Red de Epidemiología y Salud Pública (CIBERESP)-CIBER en Epidemiología y Salud Pública, Madrid, Spain; ^13^General Practitioner, 12 de Octubre University Hospital, Madrid, Spain

**Keywords:** COVID-19, mental health, women, psychiatric history, screening

## Abstract

Studies specifically designed to determine the profile of psychiatric symptoms among COVID-19 patients are limited and based on case series, self-report questionnaires, and surveys. The objective of the study was to identify and classify the neuropsychological symptoms of hospitalized COVID-19 patients during the first wave of the pandemic in one of the most important front-line tertiary hospitals from Spain, and to analyze its correlation with diagnosed mental disorders, as well as to explore potential risk factors associated with mental health problems. This observational, cohort study involved data from COVID-19 patients at the University Hospital 12 de Octubre (Madrid, Spain) from February to May 2020. First, patients underwent a semistructured phone interview (screening phase), based on the Mini International Neuropsychiatric Interview (MINI). Then the confirmation of the diagnosis (confirmation phase) was performed in patients who reported a mental disorder development or worsening. A factorial analysis was performed to identify groups of symptoms. A tetrachoric matrix was created, and factorial analysis, by a principal component analysis, was employed upon it. Factors showing values >1.0 were selected, and a varimax rotation was applied to these factors. Symptoms most frequently identified in patients were anosmia/ageusia (54.6%), cognitive complaints (50.3%), worry/nervousness (43.8%), slowing down (36.2%), and sadness (35.4%). Four factors were identified after the screening phase. The first (“anxiety/depression”) and second (“executive dysfunction”) factors explained 45.4 and 11.5% of the variance, respectively. Women, age between 50 and 60 years, duration in the hospital (more than 13 days), and psychiatric history showed significant higher levels (number of symptoms) in the factors. This study reports the factor structure of the psychiatric symptoms developed by patients with a confirmed diagnosis of SARS-CoV2 during the first wave of the COVID-19. Three item domains (anxiety, depression, and posttraumatic stress disorder symptoms) were loaded together on one factor, whereas sleep disturbance stood up as a separate factor. Interestingly, the item anosmia/ageusia was not captured by any factor. In conclusion, an increase in neuropsychiatric morbidity is expected in the upcoming months and years. Therefore, screening for early symptoms is the first step to prevent mental health problems associated with this pandemic.

## Introduction

The pandemic, caused by the severe acute respiratory syndrome virus (SARS)-CoV-2, has triggered a deep transformation of societies, with more than 240 million individuals infected worldwide (1) and 3.5 million in Spain (2). Researchers have turned their efforts in the prevention, etiopathology, risk factors, clinical symptoms, treatment, and prognosis of the coronavirus disease 2019 (COVID-19), and the scientific production has increased accordingly in just 1 year (3). Numerous studies have focused on identifying short-term physical complications derived from the disease, such as thromboembolisms or pulmonary fibrosis, for instance (4). Nevertheless, less research has been made about the psychological impact of the pandemic on patients (survivors).

In Spain, 15% of the patients have required hospitalization due to severe COVID-19 (from March 1 to July 31, 2020) (5), and about 10% of them have been admitted at the intensive care unit (ICU) (6). An early study reported that 36.4% of the hospitalized patients experienced neurologic symptoms, involving the central nervous system (especially dizziness and headache), peripheral nervous system (taste or smell impairment), and skeletal muscle injury (7). Moreover, the respiratory distress syndrome, invasive procedures, physical contentions, or the inappropriate management of sedation have been demonstrated to increase the risk of psychologic consequences and, especially, posttraumatic stress disorder (PTSD) in survivors of critical diseases (8), such as COVID-19. Diverse studies have reported additional neuropsychiatric symptoms and complications associated with the disease (including delirium, encephalopathy, olfactory disturbances, acute behavioral changes, headache, and cerebrovascular accidents) (9–11). Indeed, neuropsychiatric symptoms had also been reported after the large viral epidemics in the 19 and 20th centuries (12, 13). For example, 1 year after the SARS pandemic, the prevalence of PTSD in survivors was up to 38%. The prevalence of traumatic memories, anxiety, depression, and PTSD also raised after other coronavirus pandemics (SARS or the Middle East respiratory syndrome-CoV) (9, 14, 15). Moreover, the psychological consequences of patients surviving coronavirus epidemics are stronger than the physical ones (16).

Studies specifically designed to determine the profile of psychiatric symptoms among COVID-19 patients are limited and based on case series, self-report questionnaires, and surveys.

The objective of the present study was to identify and classify the neuropsychological symptoms of hospitalized COVID-19 patients during the first wave of the pandemic, by using screening and diagnosis confirmation, in one of the most important front-line tertiary hospitals in Spain, and to analyze its correlation with diagnosed mental disorders, as well as to explore potential risk factors associated with mental health problems.

## Methods

### Study Design

This observational, retrospective cohort study involved data from COVID-19 patients admitted at the University Hospital 12 de Octubre (Madrid, Spain) from February to May 2020 with a probable or confirmed diagnosis of SARS-CoV2 infection (by polymerase chain reaction or clinical suspicion). Patients who were not finally diagnosed of the infection during the hospitalization were excluded from the study. After discharge, all eligible patients were invited to participate in the study, and a screening phone interview was administered. Participants who reported the development or worsening of a mental disorder after the hospitalization in the screening phase were offered consultation (in-person) in the hospital to confirm the diagnosis and manage the appropriate approach. This confirmation phase (September 2020–March 2021) was developed by the same clinician who did the screening phase and was conducted through the usual diagnostic procedures in the mental health service, including a complete diagnostic interview based on DSM-5 criteria. Patients who rejected to participate or those with incomplete or unreliable information (language barrier and instrumental difficulties mostly) were excluded from the analysis.

### Evaluation of the Mental Health

After hospital discharge, two psychiatrics and one psychologist from the hospital evaluated the psychopathological status of the patients (screening phase, July–October 2020). The mental assessment of patients (screening) consisted of a semistructured phone interview, based on the Mini International Neuropsychiatric Interview (MINI) (17), and in persistent symptoms published in the available scientific literature at the time. The survey included a series of questions about the symptoms of different types of mental health problems: depression, panic attacks, generalized anxiety disorder, obsessive–compulsive disorder, PTSD, suicidal risk, substance abuse disorder (SAD) and cognitive complaints, patient sleep or feeding behaviors, and other symptoms, such as pain or anosmia/ageusia. These items were generally named as “symptoms” along the text. Answers included yes or no, and specify, when required. The original (Spanish) and translated (English) versions of the survey provided to patients are shown in Supplementary Table 1.

### Statistical Analysis

Demographic, clinical, and survey characteristics of patients and HCWs are expressed with the mean and standard deviation (SD), or with absolute and relative frequencies, when appropriate. A factorial analysis was carried out to group different variables and symptoms from the patient's survey. Given their binary categorical nature, a tetrachoric matrix was created, and factor analysis, by a principal component analysis (PCA), was employed upon it. Factors showing eigenvalues >1.0 were selected. Subsequently, a varimax rotation was applied to these factors in order to facilitate the conceptualization. Variables weighted higher than 0.5 in the axis were considered important for the factor. Constructed factors were related with demographic (gender and age), clinical (psychiatric history), and hospital stay (duration in the hospital and time until the survey). Only associations between the factors of the PCA and the risk factor of the patient showing *p* < 0.01, using a *t*-test or analysis of variance (ANOVA), were considered statistically significant. Multiple comparisons were adjusted by the Bonferroni correction. Relevant factors with more than two variables in the factorial analysis were used to determine the risk factors in patients.

## Results

### Participants

Of the 2,486 eligible patients who were initially identified, 1,694 (68.3%) were evaluable for the analysis. Causes of exclusion were not able to be contacted (*n* = 545), not willing to be interviewed (*n* = 140), not willing to collaborate in the study (*n* = 7), aged below 18 (*n* = 24), and not finally diagnosed of COVID-19 during hospitalization (*n* = 76). Table 1 shows the characteristics of the participating patients (males 52.3% and females 47.7%, with a mean age of 59.9 years).

**Table 1:** Sociodemographic characteristics of the patients.

	**Patients (*N* = 1,694)**
**Gender**, ***n*** **(%)**
Female	808 (47.7)
Male	886 (52.3)
Age, mean years (SD)	59.9 (17.0)
**Groups,** ***n*** **(%)**
≤ 50 years	492 (29.0)
50–60 years	374 (22.1)
60–70 years	315 (18.6)
>70 years	513 (30.3)
Duration in hospital, mean days (SD)	10.1 (9.4)
Time until the survey, mean days (SD)	103.0 (29.1)
Psychiatric history, *n* (%)	226 (13.3)

*SD, standard deviation*.

### Mental Problems on Coronavirus Disease 2019 Patients

Table 2 presents the symptoms most frequently identified in patients in the screening phase. These were anosmia/ageusia (54.6% of them), cognitive complaints (50.3%), worry/nervousness (43.8%), slowing down (36.2%), and depression (35.4%). A total of 175 patients (10.3%) reported none of the symptoms.

**Table 2:** Frequency of symptoms identified in patients and aggrupation into factors.

***n* (%)**	**Patients (***N*** = 1,694)**	**Factor**
Anosmia/ageusia	915 (54.6)	–
Cognitive complaints	846 (50.3)	Executive dysfunction
Worry/nervousness	740 (43.8)	Anxiety/depression
Slowing down	608 (36.2)	Executive dysfunction
Sadness	580 (34.5)	Anxiety/depression
Pain	579 (34.5)	Pain
Memory	569 (33.8)	Executive dysfunction
Insomnia	513 (30.5)	Sleep disturbances
Walking slowness	226 (13.5)	Executive dysfunction
Concentration	426 (25.3)	Executive dysfunction
Conciliation insomnia	361 (21.3)	Sleep disturbances
Maintenance insomnia	354 (20.9)	Sleep disturbances
Apathy	314 (18.7)	Anxiety/depression
Appetite alteration	302 (18.0)	Feeding/hyporexia
Pain in lower limbs	294 (17.5)	Pain
Nightmares of traumatic memories	267 (15.9)	Anxiety/depression
Anhedonia	264 (15.7)	Anxiety/depression
Headache	257 (15.1)	–
Moving slowness	226 (13.5)	Executive dysfunction
Hyporexia	180 (10.6)	Hyporexia
Bradypsychia	169 (10.2)	Executive dysfunction
Hypersomnia	162 (9.7)	–
Pain in upper limbs	159 (9.5)	Pain
Hyperphagia	148 (8.8)	Feeding
Early awakening	143 (8.4)	Sleep disturbances
Abrupt crisis scared/anxious	130 (7.72)	Anxiety/depression
Concerns that limit other tasks	118 (7.0)	Anxiety/depression
Generalized pain	102 (6.1)	Pain
Disorientation	97 (5.8)	Executive dysfunction
Bradylalia	76 (4.5)	Executive dysfunction
Death ideation	69 (4.1)	Suicidal ideation
Increase in alcohol consumption	22 (1.3)	Alcohol
Obsessions	19 (1.1)	–
Suicidal ideation	19 (1.1)	Suicidal ideation
Compulsions	17 (1.0)	Compulsions

The factorial analysis identified nine factors, of which the first seven factors explained the 87.8% of the variance (Table 3). The first factor was named as “anxiety/depression” and explained 45.4% of the variance. In this factor, items with a weight >0.50 were depression symptoms (anhedonia, sadness, apathy), generalized anxiety symptoms (worry/nervousness, concerns that limit other tasks), nightmares of traumatic memories (PTSD symptom), abrupt crisis scared/anxious, and death ideation. The second factor was conceptualized as “executive dysfunction” and explained 11.5% of the variance. It was composed of the following items (with a weight >0.50): cognitive complaints, slowing down, memory, concentration, disorientation, bradypsychia, and walking slowness. Hypersomnia was the next item with an eigenvalue under 0.5 included in this factor. The third factor was labeled as “pain” and explained 8.2% of the variance. It was composed of the variables generalized pain, pain in the upper limbs, pain in the lower limbs, and pain. Headache was the next item described with a weight under 0.5. Factors four and five explained 6.9% and 6.3% of the variance, respectively. They conformed to the dimension named as “sleep disturbances” and “suicidal ideation,” respectively. Factor six explained 4.8% of the variance and shows that alcohol consumption refusal was correlated with obsessions and suicidal ideation. The inverse correlation failed due to the low frequency of patients who indicated the consumption of alcohol (22 patients, 1.3%).

**Table 3:** Factorial analysis.

**Symptom**	**Factor1**	**Factor2**	**Factor3**	**Factor4**	**Factor5**	**Factor6**	**Factor7**	**Factor8**	**Factor9**
Anhedonia	0.88	0.23	0.19	0.13	0.00	−0.03	−0.05	0.07	0.14
Sadness	0.86	0.26	0.20	0.18	0.04	0.01	−0.01	0.03	0.19
Apathy	0.85	0.29	0.17	0.18	0.05	−0.03	−0.01	0.08	0.13
Worry/nervousness	0.85	0.17	0.11	0.28	0.03	−0.05	0.10	0.10	−0.06
Concerns that limit other tasks	0.79	0.24	0.24	0.15	0.04	−0.03	0.13	0.06	−0.03
Nightmares of traumatic memories	0.55	0.25	0.27	0.20	0.11	0.09	0.12	0.15	0.12
Abrupt crisis scared/anxious	0.53	0.23	0.14	0.22	0.23	0.10	0.19	0.17	0.13
Death ideation	0.53	0.25	0.02	0.09	0.50	−0.11	−0.04	0.11	0.18
Cognitive complaints	0.30	1.05	0.20	0.12	−0.05	−0.04	−0.07	0.05	0.06
Slowing –down	0.29	0.83	0.22	−0.03	−0.10	−0.17	−0.32	0.06	0.13
Memory	0.30	0.82	0.16	0.11	0.18	0.12	0.08	0.07	−0.02
Concentration	0.42	0.76	0.15	0.18	0.06	0.04	0.11	0.07	−0.01
Disorientation	−0.00	0.76	−0.00	0.25	0.10	0.04	0.11	−0.07	0.16
Bradypsychia	0.31	0.73	0.14	0.14	−0.24	0.04	−0.04	0.07	−0.04
Walking slowness	0.25	0.59	0.25	−0.10	0.06	−0.16	−0.35	0.04	0.16
Hypersomnia	0.20	0.38	0.11	0.16	0.21	0.05	0.04	0.10	0.10
Generalized pain	0.27	0.27	1.04	0.13	0.14	0.06	0.05	0.10	0.02
Pain in upper limbs	0.14	0.06	0.76	0.16	−0.06	−0.18	−0.18	−0.03	0.09
Pain in lower limbs	0.22	0.16	0.76	−0.05	−0.07	−0.17	−0.05	0.05	0.15
Pain	0.17	0.15	0.61	0.32	−0.14	0.19	0.03	−0.06	−0.03
Headache	0.26	0.33	0.45	0.02	0.32	0.09	0.12	0.21	−0.04
Anosmia/ageusia	0.15	0.15	0.24	0.19	0.01	0.16	0.13	0.01	−0.09
Insomnia	0.44	0.21	0.17	0.85	0.12	0.00	0.00	0.13	0.16
Early awakening	0.30	0.22	0.20	0.75	0.03	−0.13	−0.03	0.19	0.11
Conciliation insomnia	0.46	0.15	0.15	0.74	0.12	0.01	0.03	0.10	0.07
Suicidal ideation	0.27	0.08	−0.03	0.17	1.05	−0.37	−0.03	0.04	0.05
Bradylalia	0.24	0.64	0.22	0.02	−0.72	−0.11	−0.01	0.13	0.15
Increase in alcohol consumption	0.28	0.02	−0.07	0.06	−0.35	0.98	0.12	0.06	0.02
Obsessions	0.40	0.04	0.05	0.17	−0.05	−0.87	0.39	−0.04	−0.04
Compulsions	0.42	0.18	0.02	0.10	0.04	−0.14	0.92	0.06	0.16
Moving slowness	0.18	0.57	0.15	0.16	0.07	0.03	−0.85	0.01	0.12
Hyperphagia	0.15	0.07	0.03	0.18	0.03	0.05	0.02	0.98	−0.05
Appetite alteration	0.28	0.16	0.10	0.21	0.02	0.04	0.01	0.71	0.68
Hyporexia	0.32	0.20	0.11	0.16	0.04	0.03	−0.01	−0.05	0.93

*Dark red (greater than 0.50), light red (equal to 0.50), dark blue (lower than −0.50), and light blue (between −0.00 and −0.50)*.

Factors seven, eight, and nine explained 4.7%, 3.9%, and 3.3% of the variance, and constituted the dimension “compulsion” with only one item, “feeding,” including the following variables: appetite alteration and hyperphagia, and the latter, “hyporexia,” sharing the appetite alteration item with the previous factor. The remaining and most frequent item anosmia/ageusia was not appropriately explained by the factors included in the factorial analysis (final communalities lower than 0.80). Given that factors one and two grouped eight and seven variables, respectively, patients were distinguished into four groups: those who showed characteristics of both factors; patients who showed characteristics mostly from factor one or factor two, and those who showed none of the characteristics of these factors.

**Figure 1:**
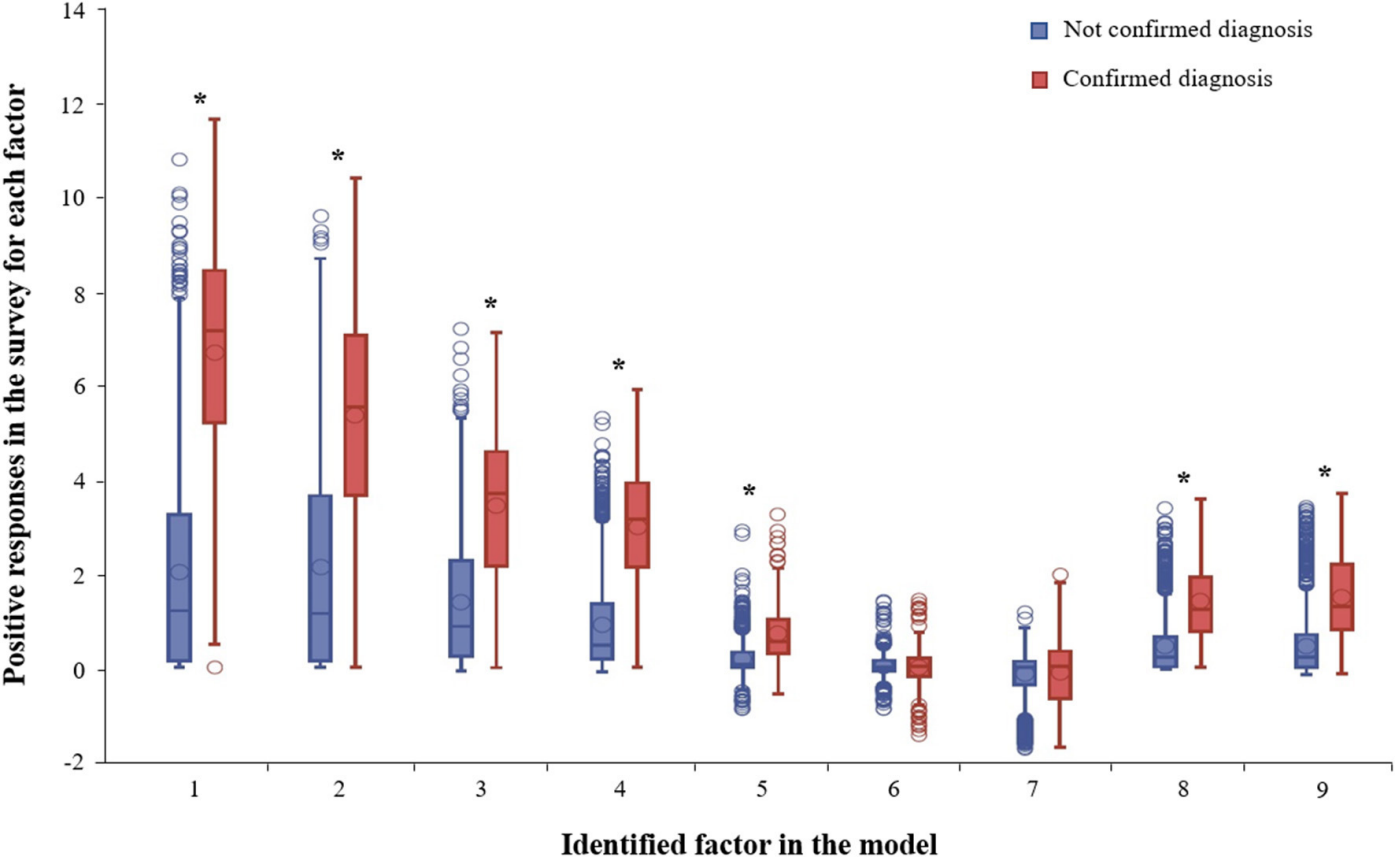
Box plot with positive responses in the survey for each factor identified in the principal component analysis considering the diagnostic confirmation. Asterisks represent statistical differences (Wilcoxon test) found between groups (*p* < 0.001).

Table 4 shows the mental disorders diagnosed in the confirmation phase, after the screening evaluation. A mental health disorder was confirmed in 14.8% of the participants who experienced significant symptoms in the screening phase. The most frequent confirmed mental problems include anxiety disorders (4.5% of them), depressive disorders (4.2%), and PTSD (2.6%). A significantly greater percentage of patients with confirmed diagnoses showed positive responses for practically all factors (except factors six and seven) than those with no confirmed diagnosis (*p* < 0.001, Figure 1).

**Table 4:** Confirmation of diagnoses from the screening phase.

	**Patients**
**Diagnosis**, ***n*** **(%)**
Not confirmed	1,429 (85.2)
Confirmed	249 (14.8)
Anxiety disorders	76 (4.5)
Depressive disorders	70 (4.2)
Posttraumatic stress disorder	43 (2.6)
Not psychopathological	23 (1.4)
Others	18 (1.1)
Toxic behaviors	9 (0.5)
Psychosis	7 (0.4)
Not available	16

### Risk Factors Associated With Mental Problems

Table 5 shows the positive responses for the first four factors, which were significantly greater in women (vs. men, *p* < 0.001 in all factors), participants with psychiatric history (vs. those with no history, *p* < 0.001 in all factors), duration of the hospital stay (especially more than 13 days, *p* = 0.002, *p* = 0.001, *p* = 0.001, and *p* = 0.021 for the factors, respectively), and age groups (mainly between 50 and 60 years, *p* < 0.001 in all factors). Regarding the time until the survey, positive responses were significantly higher for the fourth factor, especially when ≤ 92 days (*p* < 0.001).

**Table 5:** Association between patient's risk factors and main factors identified in the principal component analysis.

	**Positive responses in the survey for each factor**			
	**N**	**1 (Anxiety/** **depression/** **PTSD)**	**2 (Executive dysfunction)**	**3 (Pain)**	**4 (Sleep disturbances)**
**Gender, mean (SD)**					
Female	808	3.4 (3.0)	3.2 (2.7)	2.1 (1.7)	1.5 (1.4)
Male	886	2.1 (2.4)	2.1 (2.3)	1.3 (1.4)	0.9 (1.2)
*P*-value		<0.001	<0.001	<0.001	<0.001
**Psychiatric history, mean (SD)**					
No	1,468	2.5 (2.7)	2.5 (2.5)	1.6 (1.6)	1.1 (1.3)
Yes	226	3.9 (3.2)	3.5 (2.7)	2.2 (1.7)	1.7 (1.5)
*P*-value		<0.001	<0.001	<0.001	<0.001
**Time until the survey, mean (SD)**					
≤ 92 days	459	3.0 (3.0)	2.8 (2.7)	1.9 (1.7)	1.5 (1.5)
92–105 days	413	2.6 (2.7)	2.7 (2.5)	1.6 (1.6)	1.2 (1.2)
105–120 days	409	2.6 (2.7)	2.5 (2.5)	1.6 (1.6)	1.1 (1.3)
>120 days	413	2.6 (2.8)	2.4 (2.4)	1.7 (1.7)	1.1 (1.3)
*P*-value		0.166	0.186	0.065	<0.001
**Duration in hospital, mean (SD)**					
≤ 5 days	593	2.8 (2.9)	2.6 (2.6)	1.7 (1.7)	1.2 (1.4)
5–7 days	301	2.6 (2.8)	2.5 (2.5)	1.6 (1.6)	1.2 (1.3)
7–13 days	420	2.4 (2.7)	2.4 (2.5)	1.6 (1.6)	1.1 (1.2)
>13 days	380	3.0 (2.7)	3.0 (2.5)	2.0 (1.7)	1.3 (1.3)
*P*-value		0.002	0.001	0.001	0.021
**Age groups, mean (SD)**					
≤ 50 years	492	2.5 (2.8)	2.4 (2.5)	1.6 (1.6)	1.2 (1.3)
50–60 years	374	3.4 (3.2)	3.2 (2.8)	2.1 (1.8)	1.5 (1.5)
60–70 years	315	2.5 (2.6)	2.5 (2.3)	1.6 (1.6)	1.1 (1.2)
>70 years	513	2.5 (2.6)	2.5 (2.4)	1.5 (1.5)	1.1 (1.2)
*P*-value		<0.001	<0.001	<0.001	<0.001

*SD, standard deviation*.

## Discussion

The results obtained in this study reports the factor structure of the symptoms developed by a large sample of hospitalized patients with a confirmed diagnosis of SARS-CoV2 during the first wave of the COVID-19. The results of the factor analysis revealed that some items loaded strongly on different dimensions, above the 0.5 thresholds (Table 3). Interestingly, three item domains (anxiety, depression, and PTSD symptoms) were loaded together on one factor, whereas sleep disturbances stood up as a separate factor. One explanation could be that these two factors present two different mechanisms that underlie “anxiety–depression.” Another approach could be that “sleep disturbances” is more diagnosis oriented, while the other three item domains are oriented more toward symptoms associated with stress. A symptom dimension comprising items related to “executive dysfunction” is grouped into factor two. The items of symptoms related to pain were grouped into factor three and completed the structure of the four principal factors that accounted for 72% of the variance. Anosmia/ageusia is the most frequent symptom reported (followed by cognitive complaints, worry/nervousness, slowing down, and sadness). It is noteworthy that this neurological item was not captured by any of the factors described.

The strong convergence between the greater percentage of patients with confirmed diagnoses showing positive responses for factors one to four than those with no confirmed diagnosis was interpreted as evidence of validity in this sample.

The COVID-19 pandemic has deeply impacted on numerous facets from our lives (18). One of its consequences is the emerging of mental disorders in the general and specific populations, including COVID-19 patients. Despite the existence of literature, available studies are mainly focused on case series, self-report questionnaires, and surveys. Furthermore, available literature has provided scarce information about subclinical psychiatric symptoms by COVID-19. According to the mental health continuum model by Chen et al. (19), psychiatric symptoms are early signs of mental disorders, and individuals with severe and durable symptoms have a higher likelihood for developing them. Therefore, the early evaluation and identification of psychiatric symptoms have clinical implications for COVID-19 patients. The goals of our study were to identify and classify neuropsychological symptoms of hospitalized COVID-19 patients, study the correlation with diagnosed mental disorders, and explore the potential risk factors associated with mental health problems. The study comprised an initial screening phase and, subsequently, a diagnosis confirmation phase. The screening was conducted in COVID-19 survivors (the entire population) by hospital intern psychiatrists and psychologists. A semistructured phone interview, an *ad hoc* tool that covers a wide spectrum of psychiatric symptoms, was used. The principal component method yielded two factors that accounted for 45.4% and 11.5% of the variance, respectively. The first factor captured the items of anxiety, depression, and posttraumatic stress disorders, and the second captured the items of executive dysfunction. Factor loading was strong (all eigenvalues >0.5). These items were identified as frequent symptoms reported in patients after hospital discharge. These results are in concordance with previous literature (20–27). A study performed in China has demonstrated that insomnia, anxiety, and depressive symptoms are frequent sequels in patients after 6 months of the hospital discharge, especially in those with severe disease (28). Moreover, it has been shown that one in three survivors show executive dysfunction and mainly alterations in the attention (20). In the US, the number of diagnosed cases of anxiety, mood disorder, cognitive impairment, and insomnia increased in individuals with a diagnosis of COVID-19 (21). In Spain, ~60 and 40% of patients show moderate cognitive alterations and psychiatric morbidity, respectively, after 2 months of hospital discharge (22). Studies have also evidenced a prevalence of more than 95% for posttraumatic symptoms (23) and >30% for depression in patients with COVID-19 who have been hospitalized and stabilized (24). Studies carried out in different countries have reported a PTSD prevalence of ~20% after hospital discharge (25).

Furthermore, our results reveal that the susceptibility was higher in females and individuals aged between 50 and 60 years, after a long hospital stay (more than 13 days). Diverse studies have evidenced the higher psychological impact of the pandemic on young individuals, compared with other age groups, presumably associated with the pronounced change in their life stages (study, work, or social life) (29). Studies have also reported that younger individuals are more likely to experience loneliness than older ones (29, 30). Indeed, loneliness is strongly correlated with the development of psychological disorders (31). Despite the prevalence of confirmed mental disorder diagnosis was low, results suggest higher susceptibility in those with lifetime mental disorders, according to previously described literature. Two studies performed in Italy reported that up to 55% of the survivors have showed psychiatric sequels (PTSD, anxiety, insomnia, and obsessive–compulsive symptoms) 1 and 3 months after the infection, especially in women, young individuals, and those with lifetime mental disorders (26, 27). As documented in the literature (32–35), individuals with psychiatric illnesses are at a higher risk of aggravating their condition with the pandemic, especially among survivors (32). In a study conducted in Spain, the prevalence of cognitive impairment is higher in patients with psychiatric symptoms (22). A systematic review and meta-analysis evaluating the impact of preexisting mental illness in pandemics (including COVID-19, SARS, and influenza ones) revealed a higher development of psychiatric symptoms (mainly anxiety, depression, and insomnia) in individuals with lifetime mental conditions, compared with those without them (33). Furthermore, the authors showed a decrease in the use of psychiatric services and hospitalizations due to psychiatric events during pandemics. Similarly, Gobbi et al. (34) examining the status of 2,734 psychiatric patients worldwide and 318 from the US during the COVID-19 pandemic, concluded that more than 50% of these individuals experienced the worsening of their psychiatric conditions.

Causes associated with the development of short-term mental problems in patients may be derived from the direct effect of the virus in the central nervous system, the neuroinflammation secondary to cytokine dysregulation, the respiratory distress syndrome, the elevated prevalence of delirium, and the treatments used especially at the beginning of the pandemic (such as hydroxychloroquine, lopinavir/ritonavir, and corticoids). On the other hand, symptoms of mental health, such as the anxiety–depression–trauma complex, may be related to the confinement and other emotional stressors and traumatic memories associated with the hospitalization and the severe course of the disease (11, 13, 14, 26). It is well-known that patients remain with anosmia due to the neuronal affection induced by the virus (36). It is thought that one of the mechanisms involved in this manifestation is the disruption of olfactory neurons (37). Therefore, it may explain why that particular symptom is not eligible by any factor in the current analysis.

The main limitation of the study is derived from the methodological design in identifying symptoms associated with mental health, based on a phone interview. Although the use of specific questionnaires for each mental problem could probably strengthen the obtained results, the principal component analysis is a frequent methodology in this kind of studies, used to analyze the internal structure of a relatively large number of variables and develop predictive models. Moreover, patients were retrospectively interviewed. This may have affected the answer given by the patients. Some simplifications in the factorial analysis, useful for the result interpretation, may be poorly reproducible. Nonetheless, our observations are in line with previous studies (20–27, 32–35).

## Conclusion

This evidence points out to a presumable increase in neuropsychiatric morbidity in the upcoming months and years. Furthermore, together with the social and economic crisis, it is presumable to expect an increase in suicides in the general population and survivors, similar to that that occurred in previous coronavirus pandemics. Therefore, the multidisciplinary follow-up of patients becomes crucial to guarantee the management of their emotional consequences, with the notable relevance of mental health professionals in this scenario. In this context, screening for early signs or symptoms is the first step to improve the accuracy of early detection and prevent mental health problems. Further prospective, long-term studies, using validated questionnaires, are required to confirm our results and deepen the impact of the pandemic.

## Data Availability Statement

The raw data supporting the conclusions of this article will be made available by the authors, without undue reservation.

## Ethics Statement

Ethical approval was not provided for this study on human participants because the Ethics Committee of the center in which the study was performed considered there was no need for an evaluation since: (1) it was a retrospective study in which medical records were only reviewed; (2) no intervention was performed; and (3) the database was anonymized to preserve sensitive data from patients. Written informed consent for participation was not required for this study in accordance with the national legislation and the institutional requirements.

## Author Contributions

JM and IR reviewed the literature. GR, FR-C, and JM conceived and designed the study. IR, AJ, and CC acquired the data. DL, IR, AJ, CC, and JM cleaned and analyzed the data. JM, GR, JA, and FR-C drafted the initial version of the manuscript. All authors reviewed the initial draft and made critical contributions to the interpretation of the data and approved the manuscript.

## Conflict of Interest

The authors declare that the research was conducted in the absence of any commercial or financial relationships that could be construed as a potential conflict of interest.

## Publisher's Note

All claims expressed in this article are solely those of the authors and do not necessarily represent those of their affiliated organizations, or those of the publisher, the editors and the reviewers. Any product that may be evaluated in this article, or claim that may be made by its manufacturer, is not guaranteed or endorsed by the publisher.
