# Supplementary material for: Neuropsychological Symptom Identification and Classification in the Hospitalized COVID-19 Patients During the First Wave of the Pandemic in a Front-Line Spanish Tertiary Hospital
Source: Front Psychiatry. 2022 Mar 2;13:838239. doi: 10.3389/fpsyt.2022.838239 (PMC8924474; doi:10.3389/fpsyt.2022.838239)
Supplement: Supplementary file 1 [file Data_Sheet_1.docx]

**TRANSLATED (ENGLISH) VERSION OF THE SEMI-STRUCTURED SURVEY PROVIDED TO PATIENTS**

**1. PRESENTATION:**

“ My name is X (name of the healthcare professional) from the Department of Psychiatry of the Hospital 12 de Octubre. I am calling to you because you have been hospitalized in the hospital due to a coronavirus infection, and we are following-up all individuals who have been through this same situation. We would like to know how you are feeling and if, after being sick, you have experienced any psychological problem. To this, I would like you to answer some questions, if you agree.”

**2. EMOTIONAL VENTILATION:**

Tell me what have occurred since the initiation of the disease and you had to be hospitalized.

**3. PSYCHOPATHOLOGY:** (diverse items are evaluated)

1. SLEEP:

- *Have you experienced any sleep problem since the initiation of the disease*? (Yes / No)

If so: *The time to fall asleep, has it been higher than usual*? (Yes / No)

*Have you awoken during the night more times than usual*? (Yes / No)

*Have you awoken earlier than usual*? (Yes / No)

*Have you noticed, by contrast, if you are more sleepy than usual or if you fall asleep during the day in any situation?* (Yes / No)

2. FEEDING:

- *Have you noticed to be hungrier than usual*? (Yes / No)

3. DEPRESSION:

- *During this time,* h*ave you felt sadder, depressed, or angrier than usual?* (Yes / No)

*- Have you stopped enjoying, or do you enjoy less than usual, of the things you usually enjoy with?* (Yes / No)

*- Have you not felt like doing things?* (Yes / No)

4. PANIC ATTACK:

- *Since you got sick,* h*ave you experienced any abrupt crisis or attack in which you felt* *very anxious, scared or restless?* (Yes / No)

If so: *What did you feel or think at that time*?

(Tick the symptoms that the patient indicates)

a) Racing heart, or that the heart was pounding.

b) Wet hands.

c) Tremors or muscular twitches.

d) Shortness of breath.

e) Choking sensation or feeling of a lump in the throat.

f) Pain or disturbance in chest.

g) Nauseas or digestive disturbances, sudden diarrhea.

h) Dizziness or unsteadiness or stunning.

i) Feeling that surrounding things were unreal or unfamiliar, feeling of being separated from the body.

j) Afraid to loss the control or going crazy.

k) Afraid to be dying.

l) Some part of the body was numb or tingling.

m) Hot flushes or chills.

There is a panic attack if 4 or more symptoms are present.

5. GAD (GENERALIZED ANXIETY DISORDER):

- *Since you got sick,* h*ave you experienced to be worrier or more nervous than usual for various things?* (Yes / No)

If so: *Did you have these worries all the days*? (Yes / No)

*Did you have difficulty for controlling these worries or did they block you to perform other tasks?* (Yes / No)

6. OCD (OBSESSIVE-COMPULSIVE DISORDER):

- *Since you got sick,* h*ave you had thoughts, ideas, or impulses that you consider they are unpleasant, absurd, or distressing, that you did not want to, but you cannot stop thinking about them?* (Yes / No)

- *Have you done something repeatedly, being not able to stop it, such as wash or clean in excess, count and check out over and over, or repeat, collect, organize the things, or perform superstitious rituals?* (Yes / No)

7. PTSD (POST-TRAUMATIC STRESS DISORDER):

- *Since you got sick,* h*ave you revived, dreamt, or remembered in distress what you have experienced?* (Yes / No)

8. SUICIDAL RISK:

- *Since you got sick,* h*ave you felt like being dead or have you thought that it was better if you were dead?* (Yes / No)

*- At any time, have you wanted to hurt yourself somehow?* (Yes / No)

If so: *Have you thought how to do it or have you tried to do it at one occasion? (*Any answer determines high suicidal risk*)* (Yes / No)

9. ALCOHOL:

- *Since you got sick,* h*ave you drunk some alcohol for feeling better or falling asleep?* (Yes / No)

10. OTHER SUBSTANCES:

- *Since you got sick,* h*ave you had any product, more than once, for feeling better?* (Yes / No)

If so: *What did you take?*

11. COGNITIVE COMPLAINTS:

- *Since you got sick, is it difficult for you to remember things, for example, what you have done or where have you left something?* (Yes / No)

- *Have you noticed that is it difficult for you to be focused, for example, when you are reading, watching the TV, or speaking?* (Yes / No)

- *Have you noticed yourself as slower for walking, speak, think or moving?* (Yes / No)

If so: Specify

*Have you experienced, at any time, not knowing where you are, or which day is*? (Yes / No)

12. OTHER:

- *Since you got sick, is it more often the headache, or the pain in arm or in the whole body?* (Yes / No)

If so: Specify where the pain is.

- *Since you got sick, have you noticed a loss of smell or that the taste of foods is not as usual?* (Yes / No)

- *Have you noticed something else?*

**ORIGINAL (SPANISH) VERSION OF THE SEMI-STRUCTURED SURVEY PROVIDED TO PATIENTS**

1. **PRESENTACIÓN:**

*“Soy XXXX (nombre del profesional que realiza la llamada) del Servicio de Psiquiatría del Hospital 12 de Octubre. Le llamo porque ha estado ingresado usted en el hospital por haber tenido una infección por coronavirus y estamos haciendo un seguimiento de las personas que han pasado por esta misma situación. Queremos conocer cómo se encuentra usted y si después de enfermar ha tenido algún problema psicológico. Para saber esto, me gustaría hacerle algunas preguntas, si a usted le parece bien”*

1. **VENTILACIÓN EMOCIONAL:**

*Cuénteme qué ha ocurrido desde que empezó con la enfermedad y tuvo que ingresar en el hospital.*

1. **PSICOPATOLOGÍA**: se valoran los siguientes ítems:
2. SUEÑO:

*¿Ha tenido algún problema para dormir a partir de haber tenido esta enfermedad?* (Si / No)

Si la respuesta es SÍ:

*¿Ha tardado más de lo habitual en quedarse dormido?* (Si / No)

*¿Se ha despertado más veces de lo habitual durante la noche?* (Si / No)

*¿Se ha despertado más temprano de lo habitual?* (Si / No)

*¿Ha notado, al contrario, si tiene más sueño de lo habitual o se queda dormido durante el día, en cualquier situación?* (Si / No)

1. ALIMENTACION:

*¿Ha notado si tiene más o menos ganas de comer de lo habitual?* (Si / No)

1. DEPRESION

*¿En este tiempo se ha notado más triste, desanimado o enfadado de lo habitual?* (Si / No)

*¿Ha dejado de disfrutar o disfruta menos de las cosas que le gustaban?* (Si / No)

*¿Ha tenido menos ganas de hacer cosas?* (Si / No)

1. ATAQUES DE PANICO:

*Desde que enfermó, Ha tenido alguna crisis o ataque brusco durante el que se sintió muy ansioso, asustado o inquieto?* (Si / No)

Si la respuesta es NO, pasar al ítem 5.

Si la respuesta es SÍ:

*¿Qué sintió o pensó en esos momentos?*

(Marcar
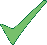
 en aquellos síntomas que refiera el paciente)

- - - - 1. el corazón acelerado, o que le latía más fuerte o más rápido,
        2. las manos húmedas,
        3. temblores o sacudidas musculares,
        4. dificultad para respirar o falta de aliento,
        5. sensación de ahogo o nudo en la garganta,
        6. dolor o malestar en el pecho,
        7. nauseas o molestias digestivas, diarrea repentina,
        8. mareo o inestabilidad o aturdimiento,
        9. sensación de que las cosas de su alrededor eran irreales o poco familiares, sensación de estar separado de su cuerpo,
        10. miedo a perder el control o volverse loco,
        11. miedo a que se estuviera muriendo,
        12. Alguna parte de su cuerpo adormecida u hormigueos,
        13. sofocos o escalofríos

Hemos acordado que si hay 4 o más síntomas la plantilla concluye Crisis de angustia.

1. TAG:

*Desde que enfermó, ¿se ha notado más preocupado o nervioso de lo habitual en usted por varias cosas?* (Si / No)

Si la respuesta es NO, pasar al ítem 6.

Si la respuesta es SÍ:

*¿Estas preocupaciones las tenía todos los días?* (Si / No)

*¿Tenía dificultades para controlarlas o le impedían concentrarse en otras tareas?* (Si / No)

1. TOC:

*Desde que enfermó, ¿ha tenido pensamientos, ideas o impulsos que consideraba que eran desagradables, absurdos o angustiosos, que no deseaba pero no podía evitar pensar?* (Si / No)

*¿Ha hecho usted algo repetidamente, sin ser capaz de evitarlo, como lavar o limpiar en exceso, contar y comprobar cosas una y otra vez o repetir, coleccionar, ordenar las cosas o realizar otros rituales supersticiosos?* (Si / No)

1. TEPT:

*Desde que enfermó, ¿ha revivido, ha soñado o ha recordado de manera angustiosa lo que le ha pasado?* (Si / No)

1. RIESGO SUICIDIO:

*Desde que enfermó, ¿ha tenido ganas de estar muerto o ha pensado que era mejor que se muriese?* (Si / No)

*En algún momento, ¿ha querido hacerse daño de alguna manera?* (Si / No)

Si la respuesta es NO a ambas preguntas, pasar al ítem 9.

Si la respuesta es SÍ a alguna de ellas: (Indicaría Riesgo de Suicidio)

*¿Ha pensado en cómo hacerlo o lo ha intentado en alguna ocasión?* (Indicaría Riesgo de Suicidio Alto, por planificación e intento previo). (Si / No)

1. ALCOHOL:

*Desde que enfermó, ¿ha bebido alcohol para sentirse mejor o conciliar el sueño?* (Si / No)

1. OTRAS SUSTANCIAS

*Desde que enfermó, ¿Ha tomado algún producto, en más de una ocasión, para sentirse mejor?* (Si / No)

*¿Qué es lo que ha tomado?”*

1. QUEJAS COGNITIVAS:

*Desde que enfermó, ¿le cuesta recordar las cosas, por ejemplo lo que ha hecho o dónde ha dejado algo?* (Si / No)

*¿Ha notado que le cueste concentrarse por ejemplo cuando está leyendo, viendo la televisión o hablando?* (Si / No)

*¿Se ha notado más lento a la hora de andar, hablar, pensar o moverse?* Si la respuesta es sí: especificar (añadir la opción andar, que no se había contemplado previamente) (Si / No)

*¿En algún momento, no ha sabido dónde estaba o día que era?*

1. OTROS:

*Desde que enfermó, ¿ha notado si le duele más la cabeza, las piernas, los brazos o todo el cuerpo?* (anotar sí con cualquiera de las cuatro opciones) (Si / No)

Si la respuesta es Sí: *¿Dónde le duele?* (abrir desplegable con cuatro opciones para clickar: cabeza, cuerpo, brazos, piernas)

*Desde que enfermó, ¿ha notado si ha perdido olfato o las comidas no le saben como siempre?* (anotar sí con cualquiera de las dos opciones) (Si / No)

*¿Ha notado algo más que no le haya preguntado?* (Si / No)
